# Patient-reported outcomes in randomized clinical trials: development of ISOQOL reporting standards

**DOI:** 10.1007/s11136-012-0252-1

**Published:** 2012-09-18

**Authors:** Michael Brundage, Jane Blazeby, Dennis Revicki, Brenda Bass, Henrica de Vet, Helen Duffy, Fabio Efficace, Madeleine King, Cindy L. K. Lam, David Moher, Jane Scott, Jeff Sloan, Claire Snyder, Susan Yount, Melanie Calvert

**Affiliations:** 1 Queen’s University, Kingston, ON Canada; 2 MRC ConDuCT Hub for Trials Methodology Research, School of Social and Community Medicine, University of Bristol, Bristol, UK; 3 Center for Health Outcomes Research, United BioSource Corporation, Bethesda, MD USA; 4 Department of Epidemiology and Biostatistics, EMGO- Institute for Health and Care Research, VU University Medical Center, Amsterdam, The Netherlands; 5 MRC Midland Hub for Trials Methodology Research, School of Health and Population Sciences, University of Birmingham, Birmingham, UK; 6 Health Outcomes Research Unit, Italian Group for Adult Hematologic Diseases (GIMEMA) Data Center, Rome, Italy; 7 Psycho-oncology Co-operative Research Group (PoCoG), University of Sydney, Sydney, Australia; 8 Department of Family Medicine and Primary Care, University of Hong Kong, Pokfulam, Hong Kong; 9 Ottawa Hospital Research Institute, Ottawa, Canada; 10 PRO Center of Excellence, Global Commercial Strategy Organization, Jansen Global Services, Warrington, UK; 11 Mayo Clinic Cancer Center, Rochester, MN USA; 12 Division of General Internal Medicine, Johns Hopkins Medicine, Baltimore, MD USA; 13 Northwestern University Feinberg School of Medicine, Chicago, IL USA

**Keywords:** Reporting, Randomized clinical trials, Quality of life, Patient-reported outcomes, Guidelines

## Abstract

**Purpose:**

To develop expert consensus on a suite of reporting standards for HRQL outcomes of RCTs.

**Methods:**

A Task Force of The International Society of Quality of Life Research (ISOQOL) undertook a systematic review of the literature to identify candidate reporting standards for HRQL in RCTs. Subsequently, a web-based survey was circulated to the ISOQOL membership. Respondents were asked to rate candidate standards on a 4-point Likert scale based on their perceived value in reporting studies in which HRQL was a study outcome (primary or secondary). Results were synthesized into draft reporting guidelines, which were further reviewed by the membership to inform the final guidance.

**Results:**

Forty-six existing candidate standards for reporting HRQL results in RCTs were synthesized to produce a 40 item survey that was completed electronically by 161 respondents. The majority of respondents rated all 40 items to be either ‘essential’ or ‘desirable’ when HRQL was a primary RCT outcome. Ratings changed when HRQL was a secondary study outcome. Feedback on the survey findings resulted in the Task Force generalizing the guidance to include patient-reported outcomes (PROs). The final guidance, which recommends standards for use in reporting PROs generally, and more specifically, for PROs identified as primary study outcomes, was approved by the ISOQOL Board of Directors.

**Conclusions:**

ISOQOL has developed a suite of recommended standards for reporting PRO results of RCTs. Improved reporting of PROs will enable accurate interpretation of evidence to inform patient choice, aid clinical decision making, and inform health policy.

## Introduction

Patient-reported outcome (PRO) data, including health-related quality of life (HRQL), from randomised clinical trials (RCTs) may be used to inform clinical decision making, health policy, and reimbursement decisions [1]. PRO data can also be used to meet patients’ information needs and to establish treatment preferences [2–6]. However, the collection of PRO data in RCTs is not without costs, including patient and investigator time, and costs associated with questionnaire administration and analysis. For PRO data from RCTs to provide value, the RCTs and PROs need to be well designed, analyzed appropriately, and reported in a way that makes the results accessible and useful to end-users. Inadequate or poor-quality reporting limits the valid application of PRO findings in clinical practice [7–9] and can limit synthesis of trial results across studies, using meta-analytic or other synthesis approaches.

Although a number of publications provide guidelines for reporting HRQL outcomes, their implementation in RCTs remains suboptimal. In a recent review of 794 trials that reported HRQL outcomes of biomedical interventions across a range of clinical areas, less than 60 % provided a rationale for the selected outcome measure or a HRQL hypothesis, 33 % did not discuss the HRQL findings within the context of other trials outcomes, and only 28 % provided information on missing HRQL data [10].

Inadequacies in trial reporting in general have been addressed through the development of the Consolidated Standards of Reporting Trials (CONSORT) Statement [11]. This is ‘an evidence-based, minimum set of recommendations for reporting RCTs’ which ‘offers a standard way for authors to prepare reports of trial findings, facilitating their complete and transparent reporting, and aiding their critical appraisal and interpretation.’[11] The original CONSORT statement and its subsequent ‘extensions’ have been widely endorsed by a number of leading international journals [12]. CONSORT standards specific to PROs, and specifically HRQL, do not currently exist. Recognizing the need for improved reporting of quality of life outcomes, the International Society for Quality of Life Research (ISOQOL) formed a Task Force to lead the development of an official CONSORT extension. This work has been undertaken in collaboration with the CONSORT executive, MRC Midland and ConDuCT Hubs for Trials Methodology Research, leading international journal editors, policy makers, and patient representatives [11, 13, 14]. In this paper, we describe the process that was undertaken by the Task Force to establish an ISOQOL consensus on standards for PRO reporting and report the resultant suite of ISOQOL-recommended standards for PRO reporting. The suite of standards is intended to inform the process of establishing a CONSORT extension for reporting PRO outcomes of RCTs based on broader stakeholder input.

## Methods

### Systematic review

A comprehensive literature search was performed in OVID Medline in April 2011 to identify papers about HRQL reporting in RCTs. Search terms were specific to Clinical Trials as Topic/or Randomized Controlled Trials as Topic/and ‘Quality of Life’/or HRQL.mp. This search yielded over 4,000 hits; consequently, the search was limited to English-language review articles from 1985 to 2010.

**Table:** Medline Search Strategy

1	Clinical trials as topic/or randomized controlled trials as topic/	222,848
2	‘Quality of Life’/or HRQL.mp	89,369
3	1 and 2	4,168
4	Limit 3 to (english language and ‘review articles’ and year = ‘1985–2010’)	1,871

Careful review of the resulting titles by two authors (MB and BB) reduced the number of potentially relevant papers to just over 300; abstracts of these papers were read and full papers obtained where the abstract illustrated potential relevance (*n* = 53). The resultant set of relevant publications was further supplemented with papers found in reference lists from seminal publications as well as those known to experts in the field. A search of the Cochrane database revealed no additional papers. All candidate standards for reporting HRQL outcome data in RCTs were abstracted from the papers identified by the comprehensive review of the literature and tabulated.

### Survey development and administration

A panel of experts forming the ISOQOL Reporting Guidelines Task Force reviewed the candidate reporting standards (‘items’) to identify redundancies and omissions and created a comprehensive list of items. An online survey was then created based on this list of items. The survey asked respondents to rate the importance of each of the items for guiding the reporting of RCT HRQL results. Two contexts were provided to the respondents: HRQL as either a primary outcome or secondary outcome in a parallel-design phase III RCT. Respondents were asked to assume that no ‘expanded’ or secondary publication focused on RCT HRQL findings would be forthcoming. Four response categories were used: (essential, desirable, optional, and rarely necessary to include). An open text field was provided to capture respondents’ comments on each item. Finally, the survey requested demographic information relating to participant expertise and experience. Respondents could return to previous items by use of a back button and modify their answers before submitting their final results. Participants were advised that through submission of their results, consent to use their data in this research was implied. Ethical approval for the survey was obtained from the University of Birmingham Ethical Review Board (ERN_11-0225). The survey was developed using Survey Monkey [15].

ISOQOL members were invited via email to participate in the survey that remained open for a 7-week period during which time two email reminders were sent. Descriptive analyses were undertaken using Survey Monkey software statistical functions and Microsoft Excel [16]. Items were ranked according to the percentage of respondents that rated the item as essential. Exploratory subgroup analyses were undertaken to assess variation in response by self-reported expertise. Participants with similar expertise were grouped as follows: HRQL assessment/psychology/sociology/predominantly in psychometrics (grouped as Psychologist/Social Scientist); frequent journal reviewer/journal editor (as Journal Editor/Reviewer); clinical trials methods/analysis (as Methodologist); predominantly clinician/clinician-scientist (as Clinician); policy/public health/regulator/health administrator (as Policy Maker). Because survey participants were able to identify more than one area of expertise, the results of these exploratory analyses are for subgroups that are not mutually exclusive.

### Development of the reporting standards

The results of the survey and free text comments were debated by the Task Force and synthesized into draft reporting standards. Items were placed into categories based on the organization of the existing CONSORT checklist of general reporting standards. Candidate items that were already contained within the main CONSORT 2010 checklist were flagged. The remaining items were grouped by the Task Force into: (1) those reporting standards recommended to be reported in any trial where HRQL is an outcome (primary or secondary) and (2) those recommended for clinical trials in which HRQL is specifically a primary outcome. Similar items were combined, resulting in a reduced draft set of reporting standards that was circulated to ISOQOL members attending the ISOQOL annual conference in Denver, CO in October 2011. Comments were invited through discussion at the Quality of Life in Clinical Practice Special Interest Group Meeting and following an oral presentation by Task Force Members. Further comments were encouraged and received by email. Feedback from the ISOQOL membership at the annual conference resulted in an important shift in the nature of the ISOQOL extension; the extension would no longer be exclusive to HRQL outcomes but rather would recommend reporting standards for all PROs. The final guidance document reflected feedback from both the ISOQOL Board and its broader membership.

## Results

### Systematic review

Fifteen papers by 14 unique first authors were identified that contained guidelines or standards for HQRL reporting in RCTs [7–9, 17–28]. From these papers, 46 HRQL RCT reporting standards were identified (Table 1). Removal of redundant items produced 40 final candidate reporting standards for use as survey items.

**Table 1:** Summary of Existing Guidance on Standards for HRQL Reporting in RCTs

	Gill 1994	Staquet 1996	Kong 1997	Revicki 2000	Lee 2000	Sprangers 2002	Movsas 2003	Efficace 2003	Wiklund 2004	Abbott 2005	Revicki 2005	Tanvetyanon 2007	Joly 2007	Street 2009	Temple 2009
*General*															
Study patient characteristics described		Y				Y					Y				
Title-explicit as to the RCT incorporating HRQL											Y				
HRQL is a stated outcome in abstract					Y						Y				
Introduction includes summary of the previous HRQL research											Y				
QOL is stated as a outcome/primary or secondary outcome					Y			Y							
Flow diagram provided/subject flow discussed		Y		Y	Y						Y				
*Instrument and admin*															
Rationale for HRQL instrument provided	Y	Y	Y		Y			Y	Y	Y	Y	Y			
Was the instrument appropriate for the stated study hypothesis?							Y							Y	
Evidence of instrument reliability		Y	Y	Y	Y	Y	Y	Y	Y	Y	Y	Y		Y	Y
Evidence of instrument validity		Y		Y	Y	Y	Y	Y	Y	Y	Y	Y		Y	Y
Adequacy of domains covered/domains of interest explicit	Y			Y	Y			Y				Y			Y
Instrument administration reported								Y		Y		Y			
Instrument responsiveness to change		Y		Y		Y	Y			Y	Y				
Copy of instrument included if not published previously		Y			Y										
Citation for HRQL instrument provided		Y			Y		Y	Y			Y				
*Analysis and reporting*															
HRQL hypothesis stated		Y		Y	Y		Y	Y			Y	Y	Y	Y	
Timing of HRQL measurement appropriate for clinical context		Y			Y	Y	Y	Y	Y		Y	Y			Y
Clinical rationale for sample size		Y			Y	Y					Y				
Power/sample size calculation		Y	Y	Y	Y*	Y	Y			Y	Y				Y
Multiple QOL items adjustment		Y		Y		Y	Y				Y		Y		Y
Missing data documented		Y		Y	Y	Y	Y			Y	Y	Y	Y		Y
Reasons for missing data discussed		Y		Y	Y	Y		Y		Y	Y		Y		
Statistical approaches taken to dealing with missing data				Y			Y			Y					
Baseline QOL status described		Y	Y		Y	Y	Y	Y		Y	Y	Y			
All HRQL data provided not just the statistically significant data				Y					Y	Y	Y	Y			
Use of appropriate statistical analysis/test of stat. sig.		Y	Y	Y			Y		Y					Y	
Post hoc analyses identified as such											Y				
Survival difference accounted for						Y				Y					
Clinical significance addressed/MID stated		Y		Y	Y	Y	Y	Y	Y	Y	Y	Y			Y
Generalizability of the results described				Y							Y				
Mode of administration stated/methods of collecting data		Y			Y				Y		Y				
Blinding of investigators and participants			Y								Y				
*Other*															
Procedures for quality control		Y		Y	Y		Y		Y						Y
Compliance monitoring			Y	Y			Y								
Pilot testing			Y												
Authors provide composite score for quality of life	Y														
Were patients asked to provide their own global rating for QOL?	Y														
Was overall quality of life distinguished from HRQOL?	Y														
Were patients invited to supplement the items in the instrument?	Y														
Were the items incorporated into the final rating?	Y														
Were patients asked which items were personally important?	Y			Y											
Were these important ratings incorporated into the final rating?	Y														
Designated symptom or QOL domain as a primary endpoint													Y		
Proportion achieving a pre-defined palliative response													Y		
Estimates of the duration of palliative response													Y		
Discussion of the limitations of the results													Y		

* When HRQL primary endpoint

### Survey results

**Fig. 1:**
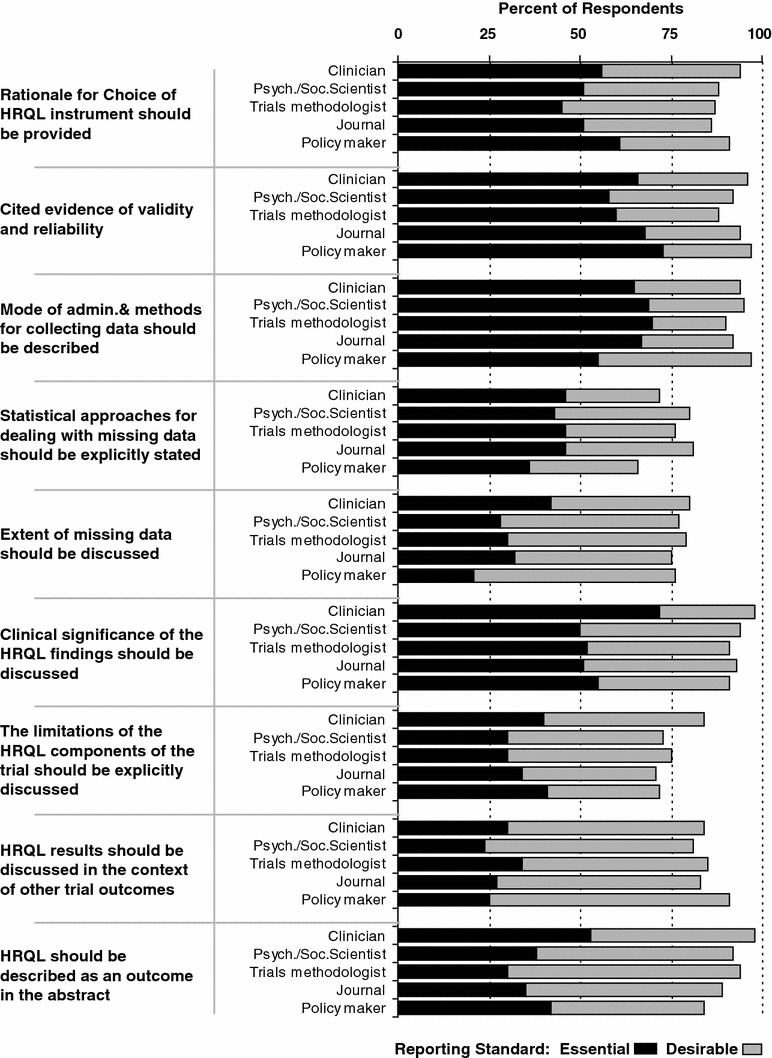
Survey response by participant expertise for selected reporting standards

There were 161 respondents to the survey, of which 144 (97 %) were confirmed current members of ISOQOL. The survey link was potentially received by an estimated 480 members of ISOQOL. Although the number of invitations actually received could not be determined, the ratio of invitations to respondents implies a response rate of approximately 30 %. The majority (63 %) of respondents had an excess of 10 years experience in HRQL research (Table 2). Thirty-six of the 40 items (90 %) were rated as ‘essential’ by at least half of the respondents, 27 (68 %) were rated as ‘essential’ by at least two-thirds of respondents, and all items (100 %) were rated as either ‘essential’ or ‘desirable’ by over three quarters of respondents (Table 3). These rankings changed when HRQL was a secondary outcome; with 12 items (30 %) then rated as ‘essential’ by over one-half of respondents, and 26 (65 %) rated as either ‘essential’ or ‘desirable’ by over three quarters of respondents (Table 3). Results were fairly consistent across subgroups (by expertise) in exploratory analyses. Notably however, a higher proportion of clinicians rated the reporting of the clinical significance of results as ‘essential’ (Fig. 1).

**Table 2:** Survey respondent demographics

	*N* (%)
*Participant expertise n* *=* *149*	
HRQL assessment/psychology/sociology	105 (70.5)
Frequent journal reviewer	71 (47.7)
Clinical trials methods/analysis	67 (45)
Predominantly in psychometrics	47 (31.5)
Predominantly as clinician—scientist	38 (25.5)
Policy/public health	31 (20.8)
Predominantly as clinician	19 (12.8)
Journal editor	19 (12.8)
Patient perspective	9 (6)
Regulator/health administrator	3 (2)
Other*	9 (6)
*Career experience in HRQL research or related activities n* *=* *148*	
Currently undergrad/PhD student	2 (1.4)
Currently post doc	11 (7.4)
<5 years experience	13 (8.8)
5–10 years experience	29 (19.6)
>10 years experience	93 (62.8)

* Other: PhD nursing student spent the past year critiquing articles, including studying CONSORT and other guidelines; Conducting literature reviews of treatment effects and HRQL endpoints; Systematic reviews and comparative effectiveness research; Associate Editor; Expertise in economic evaluations; Academic interest in performance and standards for interventions and implementation; Nurse Epidemiologist; Expertise in clinimetrics/psychometrics, hardly no experience in trials; Expertise in QOL assessment in communication disorders; Systematic reviewer Statistical editor; HRQL Instrument development and promulgation of use of these measures

**Table 3:** Survey responses

Item	When HRQL is a primary outcome						When HRQL is a secondary outcome					
	Rank	*n*	Percentage response				Rank	*n*	Percentage response			
			Essential	Desirable	Optional	Rarely necessary			Essential	Desirable	Optional	Rarely necessary
HRQL should be identified as an outcome in the abstract	1	160	96.9	2.5	0.6	0.0	18	157	38.9	53.5	7.6	0.0
The study patient characteristics should be described	2	160	95.0	3.8	1.3	0.0	1	158	85.4	12.7	1.9	0.0
The mode of administration of the HRQL tool and the methods of collecting data (e.g., telephone, other) should be described	3	150	90.0	8.0	2.0	0.0	3	150	65.3	27.3	6.0	1.3
The domains of interest should be explicitly stated and be appropriate for the disease/treatment context	4	154	89.6	9.1	0.6	0.6	10	153	56.2	37.9	5.2	0.7
The clinical significance of the HRQL findings should be discussed	5	150	88.7	11.3	0.0	0.0	11	150	52.7	39.3	7.3	0.7
The baseline HRQL scores of study participants should be described	6	151	86.8	10.6	2.6	0.0	8	152	58.6	28.9	10.5	2.0
There should be evidence of appropriate statistical analysis and tests of statistical significance for each HRQL hypothesis tested	7	148	85.8	12.2	0.7	1.4	7	148	59.5	33.1	6.1	1.4
The HRQL hypothesis should be stated and should specify the relevant HRQL domain(s)	8	153	85.0	13.1	1.3	0.7	17	153	39.2	47.7	11.8	1.3
Reporting of who is blinded to treatment allocation in the trial should be provided	9	150	84.0	10.0	4.7	1.3	2	149	65.8	24.8	8.1	1.3
The status of the HRQL outcome as either a primary or secondary endpoint should be stated	10	160	83.1	15.6	1.3	0.0	–	–	–	–	–	–
There should be cited evidence of instrument validity	11	153	83.0	13.7	2.6	0.7	5	152	61.2	31.6	5.9	1.3
The rationale for choice of the HRQL instrument used should be provided	12	153	81.0	17.0	2.0	0.0	13	153	49.0	38.6	11.1	1.3
There should be cited evidence of instrument reliability	13	154	80.5	16.9	1.9	0.6	6	154	59.7	31.8	6.5	1.9
HRQL hypotheses should specify time points at which the HRQL outcomes will be compared	14	153	80.4	17.0	2.6	0.0	9	152	57.2	32.2	9.9	0.7
The introduction should contain a summary of HRQL research that is relevant to the RCT	15	159	79.2	15.7	4.4	0.6	35	157	21.0	53.5	23.6	1.9
The authors should discuss the limitations of the HRQL components of the trial explicitly	16	149	77.9	19.5	2.7	0.0	25	150	31.3	43.3	20.7	4.7
There should be a clinical rationale provided for the sample size (e.g., anticipated effect size)	17	149	76.5	21.5	1.3	70.0	34	149	23.5	47.7	20.1	8.7
A citation for the original development of the HRQL instrument should be provided	18	154	76.0	18.8	4.5	0.6	4	154	64.3	26.6	7.8	1.3
Statistical approaches for dealing with missing data should be explicitly stated	19	149	75.8	18.1	6.0	0.0	16	151	43.7	35.1	17.9	3.3
There should be a power/sample size calculation relevant to the HRQL outcome	20	153	75.8	17.6	5.2	1.3	36	152	17.8	41.4	24.3	16.4
Any post hoc analyses of HRQL data should be identified	21	146	74.7	19.9	3.4	2.1	12	145	51.0	33.8	13.1	2.1
The intended HRQL data collection schedule should be provided	22	151	71.5	23.2	4.6	0.7	14	151	48.3	36.4	13.2	2.0
The generalizability of the HRQL results should be described	23	148	69.6	27.7	2.7	0.0	21	148	36.5	42.6	18.9	2.0
The extent of missing HRQL data should be documented at each time point	24	151	69.5	21.9	7.3	1.3	26	151	30.5	45.0	21.2	3.3
Hypotheses should specify the direction of change of HRQL outcomes	25	152	69.1	23.7	5.9	1.3	15	152	45.4	39.5	13.2	2.0
The title of the paper should be explicit as to the RCT including an HRQL outcome	26	159	67.9	23.9	7.5	0.6	39	157	9.6	45.9	38.9	5.7
There should be cited evidence of instrument responsiveness to change	27	150	66.7	26.0	6.7	0.7	20	150	36.7	48.0	13.3	2.0
The manner in which multiple comparisons have been addressed should be provided	28	149	65.8	29.5	2.7	2.0	19	150	38.0	43.3	16.0	2.7
The HRQL results should be discussed in the context of the other clinical trial outcomes	29	151	62.9	31.8	5.3	0.0	31	150	27.3	54.0	16.7	2.0
The reasons for missing HRQL data should be discussed	30	150	62.7	31.3	4.7	1.3	32	150	24.0	49.3	22.7	4.0
Results should be reported for all HRQL domains and items identified by the reference instrument (i.e., not just those that are statistically significant)	31	150	62.7	25.3	9.3	2.7	24	150	32.0	42.7	20.0	5.3
Evidence should be provided that the reported HRQL results were prespecified in the protocol	32	143	61.5	25.9	8.4	4.2	28	144	29.9	42.4	18.8	9.0
The data collection schedule should be justified as being appropriate for the clinical context	33	148	59.5	32.4	6.1	2.0	22	147	33.3	45.6	16.3	4.8
A flow diagram or a description of the allocation of participants and those lost to follow-up should be provided for HRQL specifically	34	151	58.9	25.8	11.9	3.3	38	150	12.7	41.3	32.0	14.0
Where survival is a relevant trial outcome, HRQL analysis should account for survival differences between treatment groups	35	138	55.1	36.2	6.5	2.2	23	138	34.1	40.6	19.6	5.8
The time window for valid HRQL responses should be specified	36	147	52.4	36.7	10.2	0.7	27	145	30.3	46.2	21.4	2.1
Quality control procedures for HRQL data should be provided	37	146	48.6	35.6	12.3	3.4	29	146	28.1	36.3	27.4	8.2
The proportion of patients achieving pre-defined responder definitions should be provided	38	144	47.2	30.6	18.8	3.5	33	143	23.8	42.0	28.0	6.3
A copy of the instrument should be included if it has not been published previously	39	153	44.4	31.4	16.3	7.8	30	153	28.1	28.1	32.0	11.8
The report should state how HRQL data collection protocols were monitored	40	149	39.6	36.9	17.4	6.0	37	147	17.7	41.5	26.5	14.3

### ISOQOL-recommended PRO reporting standards for randomized clinical trials

The results of the survey and feedback from the ISOQOL membership were used to develop a suite of reporting standards. While the survey focused specifically on the development of standards for use in studies in which HRQL is an outcome, these standards were acknowledged to be appropriate for PROs in general, outcomes such as single-item symptom evaluation, patient-reported measures of utility, or assessment of patient adherence and satisfaction (Table 4) [29].

**Table 4:** ISOQOL-recommended PRO reporting standards for randomized clinical trials

Reporting standard category	Original CONSORT guidance 2010	Additional standards recommended for all studies with a PRO	Additional standards recommended for studies in which the PRO is a 1° outcome
		(Regardless of whether the PRO is a 1° or 2° outcome)	
Title and abstract	1a. Identification as a randomised trial in the title		The title of the paper should be explicit as to the RCT including a PRO
	1b. Structured summary of trial design, methods, results, and conclusions	The PRO should be identified as an outcome in the abstract	
Introduction, background, and objectives	2a. Scientific background and explanation of rationale		The introduction should contain a summary of PRO research that is relevant to the RCT
	2b. Specific objectives or hypotheses	The PRO hypothesis should be stated and should specify the relevant PRO domain(s) if applicable	Additional details regarding the hypothesis should be provided, including the rationale for the selected domain(s), the expected direction(s) of change, and the time points for assessment
*Methods*			
Outcomes	6a. Completely defined pre-specified primary and secondary outcome measures, including how and when they were assessed	The mode of administration of the PRO tool and the methods of collecting data (e.g., telephone, other) should be described	A citation for the original development of the PRO instrument should be provided
	6b. Any changes to trial outcomes after the trial commenced, with reasons	The rationale for choice of the PRO instrument used should be provided	Windows for valid PRO responses should be specified and justified as being appropriate for the clinical context
		Evidence of PRO instrument validity and reliability should be provided or cited	
		The intended HRQL data collection schedule should be provided	
		PROs should be identified in the trial protocol; post hoc analyses should be identified	
		The status of PRO as either a primary or secondary outcome should be stated	
Sample size	7a. How sample size was determined		There should be a power/sample size calculation relevant to the PRO based on a clinical rationale (e.g., anticipated effect size)
	7b. When applicable, explanation of any interim analyses and stopping guidelines		
Statistical methods	12a. Statistical methods used to compare groups for primary and secondary outcomes	There should be evidence of appropriate statistical analysis and tests of statistical significance for each PRO hypothesis tested	The manner in which multiple comparisons have been addressed should be provided
	12b. Methods for additional analyses, such as subgroup analyses and adjusted analyses	Statistical approaches for dealing with missing data should be explicitly stated, and the extent of missing data should be stated	
*Results*			
Participant flow (a diagram is strongly recommended)	13a. For each group, the numbers of participants who were randomly assigned, received intended treatment, and were analyzed for the primary outcome	A flow diagram or a description of the allocation of participants and those lost to follow-up should be provided for PROs specifically	
	13b. For each group, losses and exclusions after randomisation, together with reasons	The reasons for missing data should be explained	
Baseline data	15. A table showing baseline demographic and clinical characteristics for each group	The study patients’ characteristics should be described, including baseline PRO scores	
Outcomes and estimation	17a. For each primary and secondary outcome, results for each group, and the estimated effect size and its precision (such as 95 % confidence interval)		The analysis of PRO data should account for survival differences between treatment groups if relevant
			Results should be reported for all PRO domains (if multi-dimensional) and items identified by the reference instrument (i.e., not just those that are statistically significant)
			The proportion of patients achieving pre-defined responder definitions should be provided where relevant
	17b. For binary outcomes, presentation of both absolute and relative effect sizes is recommended		
*Discussion*			
Limitations	20. All important harms or unintended effects in each group	The limitations of the PRO components of the trial should be explicitly discussed	
Generalizability	21. Generalizability (external validity, applicability) of the trial findings	Generalizability issues uniquely related to the PRO results should be discussed, if applicable	
Interpretation	22. Interpretation consistent with results, balancing benefits and harms, and considering other relevant evidence	The clinical significance of the PRO findings should be discussed	
		The PRO results should be discussed in the context of the other clinical trial outcomes	
*Other information*			
Protocol	24. Where the full trial protocol can be accessed, if available		A copy of the instrument should be included if it has not been published previously

## Discussion

Synthesis of a systematic review of the literature and survey of the ISOQOL membership has informed the development of ISOQOL reporting guidelines for PROs in the primary publication of RCTs. More specifically, we provide reporting standards for RCTs reporting any PRO outcome and make additional recommendations for those trials in which PROs are the primary outcome. This work will be used to inform an official CONSORT extension for PRO reporting in RCTs.

While current CONSORT guidelines for trial reporting include several standards that apply to HRQL and PRO endpoints, the Task Force felt that further clarification was needed [11]. For example, the 2010 CONSORT guidance states that ‘a table showing baseline demographic and clinical characteristics for each group should be reported.’ However, neither the guideline nor the explanatory document explicitly recommends that publications report baseline values relating to the primary or secondary PRO endpoints with a measure of their variability. There is also a lack of clarity about the appropriate level of detail required for reporting missing outcome data, which is particularly critical when interpreting PRO trial endpoints.

The new ISOQOL reporting guidelines have important implications beyond improved documentation of trial outcomes if they also act to influence the design of future trials with PRO measures. Evidence for this type of impact is apparent in other areas; since the publication of the original CONSORT guidance, there is now work being undertaken to provide trialists and clinicians with standards for trial protocol development [30]. Future international collaborations among stakeholder groups could include similar efforts for informing trial design with respect to PROs.

The reality of journal word limits may constrain comprehensive reporting of all of the ISOQOL-recommended standards, particularly when the PRO is a secondary study outcome. A recent review of HRQL reporting in clinical trials across a range of clinical areas showed that HRQL was a secondary outcome in 75 % of studies (594/794) [10]. Without transparent reporting of these secondary outcomes, it may be impossible for readers or consumers to assess the quality of the results and identify any potential bias. Journal web appendices are a relatively new option that may, in part, alleviate this problem.

The process used to establish PRO reporting standards for RCTs has a number of strengths. The candidate reporting standards were identified through a formal systematic review, and the guidance was developed through a comprehensive iterative process with multiple opportunities for feedback from the experts within the ISOQOL membership who have a collective, extensive international experience in this field. A modified Delphi approach was used to assess member opinions and to reach consensus on a final list of reporting standards; this approach is consistent with the development of other CONSORT guidelines [31]. The consistency of endorsement of each standard across different areas of respondents’ expertise is an indication of the reliability of the survey data. One notable, but perhaps not surprising, exception to this consistency is that a higher proportion of clinicians rated reporting of the clinical significance of results as essential, demonstrating the potential importance of this item for translation of HRQL results into practice.

The interpretation of the survey results and the consensus recommendations are limited in some ways including the use of the online survey method to poll ISOQOL members. Although we attempted to invite all ISOQOL members to respond, it is unknown how many members actually received or viewed the email, and an accurate response rate cannot be calculated. Likewise, the ‘view rate’ of email invitations cannot be calculated due to the anonymous nature of the research method [32]. Further, it is likely that some ISOQOL members felt that they did not have sufficient expertise or interest in this academic topic to respond, and regrettably, we did not allow such a response option in our design. Likewise, it is possible that some respondents with only passing interest in the topic may have completed the survey without sufficient knowledge or experience in designing, analyzing, reporting, or interpreting RCTs. With regard to the latter possibility, however, we note that the majority of respondents did report having significant expertise in HRQL research. Again, the consistency of responses across expertise groups suggests reliability of the findings. Although there was clearly underlying variation in ISOQOL member opinions, particularly when HRQL is a secondary outcome, the systematic approach to the data by the Task Force led to final recommended standards that are in keeping with the majority view of the member survey results for each standard considered in the survey. Finally, while the literature search and survey focussed on HRQL standards, rather than all PROs, the consensus process endorsed the expanded scope.

### Conclusions and implications

The results of this literature review and survey enabled an ISOQOL Task Force to arrive at consensus regarding recommended standards for reporting PROs in publications of randomized clinical trials. These recommendations were developed with input from the membership and endorsed by the ISOQOL leadership and have now been used in conjunction with information from other key stakeholders to inform a Consensus meeting and the development of a new CONSORT extension to guide the reporting of PROs [11, 31]. Knowledge transfer activities including endorsement from major journals will be crucial to the success of the CONSORT extension. This guidance will need to be evaluated with respect to its impact on the quality of PRO reporting, and we note that no formal evaluation of the past published recommended reporting standards has been undertaken. In keeping with promoting effective knowledge translation, the ISOQOL standards themselves will be available through the ISOQOL website and related publications and will serve as continued guidance for RCT reporting that reflects the ISOQOL consensus.

In sum, consumers of PRO data (including HRQL), who may be healthcare information providers, clinicians, policy makers, and patients themselves, should demand high-quality, well-reported data from RCTs. Likewise, organizations that fund clinical trials research should also be aware of issues relevant to PRO reporting and should require standardized reporting to facilitate use of PRO data in clinical practice. These consensus guidelines are an important component of establishing standards that will lead, through effective knowledge translation processes, to improved reporting practices.
